# A solid base for scaling up: the structure of numeration systems

**DOI:** 10.1098/rstb.2024.0207

**Published:** 2025-10-20

**Authors:** Jean-Charles Pelland, Simon J. Greenhill, Mary Walworth, Andrea Bender

**Affiliations:** ^1^ Department of Psychosocial Science, University of Bergen, Bergen, Norway; ^2^ School of Biological Sciences, The University of Auckland, Auckland, New Zealand; ^3^ Department of Linguistic and Cultural Evolution, Max Planck Institute for Evolutionary Anthropology, Leipzig, Germany; ^4^ Centre National de la Recherche Scientifique, UMR 8563, Aubervilliers, France

**Keywords:** cognition, culture, evolution, embodied tools, numerals, numerical cognition

## Abstract

While numeration systems are found in almost every human society, they also vary strikingly around the globe. One important feature of many systems is being structured around a base. The presence, format and size of a base have implications for how representations of numbers are composed, conceptualized and used. The numerical cognition literature is rife with claims about which bases prevail, with sweeping generalizations on their origins and evolution. Yet these claims are rarely scrutinized, and research on numeration systems is plagued by a surprising lack of consensus on what a base *is*. This theme issue brings together scholars from the cognitive, social and behavioural sciences for a comprehensive overview of bases, aimed at creating common ground for communication and collaboration across disciplines. Contributions include (i) proposals for conceptual clarification and consistency, aided by a tool for visualizing system structure; (ii) reports on how bases are realized across different representational formats, cultures and time, in search of patterns and evolutionary trajectories; and (iii) analyses of cognitive implications and cultural imprints of bases. The main goal of this endeavour is to help build an integrative synthesis of both theorizing and findings on the cultural evolution of a key cognitive tool.

This article is part of the theme issue ‘A solid base for scaling up: the structure of numeration systems’.

## 1. Who’s on base?

When experiencing car trouble, those of us who are not mechanics typically come to an unfortunate realization after popping the hood: knowing how to use something does not translate to knowing how it works. This chasm between *knowing-how* and *knowing-that* applies to physical tools and technologies such as cars and their engines, but it also applies to cognitive technologies such as numeral systems and their base. While most of us know how to count and calculate, few could list the rules that govern this ability, and even fewer could explicitly describe the precise role played by the base of the numeration systems (also known as numeral systems) we use. It might not sound like this is a big deal: some of us are mechanics, others are academics. So, some can tell us how cars work, and others can tell us how numeration systems work. Sadly, the situation is not quite so simple: while academics from a wide diversity of domains do study numerical cognition, the base of a numeration system, which is the compositional motor running our numerical abilities, has yet to receive any systematic treatment.

To see how this is a problem, we need only compare this situation with that of language and its key components. Chomsky’s investigation into the concept of a grammar contributed to the demise of behaviourism and catalysed the cognitive revolution that followed. Decades later, the twentieth century’s linguistic turn may have cooled a bit, and Chomsky’s influence is certainly not what it used to be, but the investigation into our ability to compose sentences out of words is still very much alive and well. Universities across the globe have dedicated linguistics departments, and specialized linguists travel the world, sampling grammars from Malawi to Micronesia, recording lesser-known constructions that might help find patterns that expose the roots of our compositional linguistic abilities.

The same cannot be said for our ability to form complex numerals. Sure, after its timid beginnings in the late 1800s with the work of Jevons and Cattell, the field of numerical cognition is now thriving, with a dedicated journal, thousands of articles and many collections (e.g. [1–4]) uncovering the mechanisms behind our numerical abilities. Crucially, however, targeted investigation into the specific compositional role played by a base in our numerical abilities is essentially non-existent, despite the undeniable importance that numeracy has in our lives. Aside from a few mentions of bases in texts dealing with numerical notations and lexical (i.e. linguistic) numerals, where bases are typically considered central parts of numeration systems, no books, no conferences, and not even a single article seems to have been dedicated to the concept of the base of a numeration system itself—until now.

To get a better idea of why bases might be more important than their lack of academic attention suggests, consider the following claim from the classic treatise by nineteenth century mathematician Levi Conant on the origins of numbers: ‘Without the establishment of some base any system of numbers is impossible’ [5, p.101]. While Conant lists bases of various lexical systems across the world, he does not tell us what bases are. He does, however, provide hints as to what the base of a lexical numeration system does—e.g. a base offers its users a ‘well-defined milestone’ with which individuals can keep track of where they are in their counting routines. Instead of always having to refer back to zero, we can use our base as a reference point in the land of numbers to get our bearing on numerical magnitude: every time we reach a new number, all we need to do is ‘indicate the distance [we have] progressed beyond [our] base, and not the distance from [our] original starting-point’ [5, p.101].

Many linguists, including from descriptive and typological traditions, have also worked on bases, with a few offering definitions to help situate these in their work on lexical numerals. As is extensively documented in Barlow’s [6] contribution to our theme issue, these linguistic definitions vary substantially, although they share a general conception of bases as numerical values used in forming numerals.

Outside of linguistics, the study of notation has offered a more homogeneous, precise characterization of bases that includes reference to powers of a particular number. Chrisomalis’ [7] extensive taxonomy of numerical notations, for instance, defines a base as ‘a natural number B in which exponents of B are specially designated’ (p.4). Similarly, Widom & Schlimm’s [8] taxonomy of notations sees a system’s base as a number b whose powers b^0^, b^1^, b^2^ … b^*n*^ receive special significance, often in the form of their own signs, with the effect of collecting all previous lesser units into a new, greater unit’ (p.158).

Such wide divergence between definitions is a problem: terminological confusion and crosstalk pose serious challenges for any cross-disciplinary research programme. This became painfully clear to attendees of a meeting for Quanta, a European Research Council (ERC)-funded interdisciplinary project aiming to investigate the origins and evolutionary trajectories of numeration practices and tools. At this meeting, cognitive, social and behavioural scientists had come together to discuss how to compare numeration systems across representational formats (i.e. body-based, verbal/lexical, material and notational)—only to discover that a lack of consensus on what the word ‘base’ and its derivatives mean and the concomitant lack of consistency in how datasets were coded and categorized were major obstacles to this endeavour.

Improving this situation was the key motivation for this theme issue.

## 2. A solid base to build on

In order to create common conceptual ground for communication and collaboration across fields, we solicited contributions from a wide range of disciplines, each targeting base-relevant issues from distinct perspectives, including (in alphabetical order) anthropology, cognitive science, computational modelling, history of mathematics, linguistics, mathematics, philosophy and psychology. The authors who answered our call all take essential steps towards our joint goal. Some are doing so by laying the groundwork with integrative syntheses of base concepts or by systematic overviews of how bases are realized across representational formats. Others make inroads regarding empirical questions on how frequent, variable and evolutionary stable bases are, or how they affect the cognitive representation and processing of numerical information. Yet others explore new territory on what this tells us about the cultural evolution of a crucial component of our modern cognitive lives.

The contributions to this theme issue are loosely grouped into three clusters, beginning with those that tackle the challenges of diverging concepts across disciplines (§2a), followed by articles exploring numeral bases and related structuring components across modalities, cultures and time (§2b) and concluding with those that investigate the cognitive implications of bases, as well as their cultural imprint and embedding (§2c).

### (a) Conceptions and challenges across disciplines

An important step towards common conceptual ground is clarifying where divergences lie and what can be done to bring differing conceptions of bases together. Barlow [6] provides this clarification in the field of linguistics by detailing the various ways in which linguists have understood the concept of a numeral base and organizing these into a terminological typology of ‘bases’. This allows him to compare and contrast how bases are seen in lexical systems in terms of their frequency and regularity of use, as well as in relation to the operations that apply to them, thus paving the way for comparative analyses that can uncover recurring structural patterns in numeral systems across the world’s languages.

The way that linguists have framed bases is difficult to reconcile with how mathematicians and those studying notations have approached them. In his contribution to this theme issue, Pelland [9] argues that neither framework can be extended to the other, and that a separate conceptual scheme must be introduced to capture relations between compositional tools of numeration systems across all representational formats. To help unite the real-world concerns in linguistics and the idealistic approaches to notational systems, Pelland’s contribution introduces the notion of a compositional anchor as any number conventionally used as a counting unit, of which bases are a special case.

Of course, on top of their incarnation in notations and languages, bases manifest themselves in wildly different ways of representing numbers, which makes it difficult to analyse and compare compositional elements across representational formats. Schlimm [10] offers a novel framework geared towards representing the structure of numeration systems visually with the help of images of (an abstract representation of) an abacus, where each column represents the value of a power of the base or sub-base of the system. With this representation, we can easily compare systems in terms of their structures and pinpoint irregularities—irrespective of the format in which they are implemented.

While the terminological conventions agreed upon by our contributors (codified in Pelland’s [11] *Glossary*) may only mark the beginning of a multidisciplinary conceptual framework for talking about compositionality in numerals and numerical cognition more broadly, the above articles advocating them highlight the need to converge on shared concepts, labels and instruments at the intersection of previously distinct traditions. One of the options that such convergence offers is to compare systems also across formats and, for instance, investigate whether and how bases manifest themselves in different ways depending on those formats.

Chrisomalis [12] is pioneering this type of investigation with a contribution aimed at explaining how two widespread types of numeration systems—notational and lexical systems—are structured by their modality (framed here in a technical sense that integrates the sensory medium in which numerals are perceived as well as the structural rules that govern their use and production). He argues that our brain-bound cognitive systems impose modality-specific constraints on how we process content presented in cognitive tools: visual and auditory stimuli are just not processed in the same way. Chrisomalis shows how such modality-specific constraints are reflected in how properties of graphical systems such as notations differ from those of lexical systems, whose properties are inherited from the vocal–auditory channel. Each modality will handle ambiguity avoidance, phrase ordering and conciseness of composite numerals differently. Chrisomalis’ meticulous analysis of specific features of written notations versus spoken lexical numerals is a perfect example of what can be achieved when taking the notion of distributed cognition [13] seriously. Seeing cognition as being distributed means that cognitive systems can include both brain-bound and extra-cranial objects, so that the system’s behaviour is the result of interactions between the brain and objects in its environment. Chrisomalis reflects this approach to the mind by explaining features of the cognitive tools in terms of the way in which our brains have to interact with them.

### (b) Bases and related structuring components across modalities, cultures and time

Even when we do make progress towards terminological consistency, definitions only give us an *idea* of what we are talking about. However, knowing that something is a car engine does not help most of us know how it works. So, even if we agree on what bases are, we are just getting started in understanding how they work, where they come from and what sort of effects they have on our minds and cultures. To tackle these gaps in our knowledge, we need to address another critical challenge faced when investigating bases of numeration systems: their diversity. Numerals come in a plethora of representational formats and vehicles that are implemented across many sensory modalities. Thousands of languages each have their own numeral system(s), in addition to close to 200 written notations and an unknown number of body-based and material systems, including knot-based *quipus*, *yupanas*, wooden counting rods and pebbles organized into abacuses.

This stunning diversity raises the question of whether and how all these systems are related. Can we use their compositional structures and the associated bases or anchors to pinpoint ancestral relations between systems or to home in on potential origins of numeration practices and tools, as is done in phylogenetic reconstruction (for an example see [14]?

Unfortunately, not all modalities and representational formats have been equally well investigated and documented. Lexical and notational systems in particular have received markedly more attention than body-based representations. Even among the former—and despite a substantial amount of work already done (e.g. [15–20])—many languages still await proper documentation of their numerals’ compositional structure. The second cluster of this theme issue combines articles offering a more in-depth look at specific systems that are unique and novel for a variety of reasons with contributions offering a more comprehensive bird’s-eye view on larger samples of related systems. Collectively, they expose patterns of commonalities and differences in structuring components across modalities, cultures and time, and thereby help to reconstruct shared origins and evolutionary trajectories.

The cluster begins with two articles focusing on systems with restricted extent, which contain only very few (if any) number words and often lack compositional structure. The most famous examples are perhaps the Amazonian languages Munduruku and Pirahã. Research on such languages has had tremendous impact on the field of numerical cognition, as well as for theories of language (e.g. by inspiring debates on whether or not recursion is a linguistic universal, as in [21,22]) since they offer a unique glimpse into quantitative cognitive tools that are not influenced by western cultural practices.

Zariquiey *et al.* [23] take readers in southwest Amazonia for an in-depth investigation of how Headwater Pano languages denote discrete quantities. Carefully documenting and classifying the quantity-related expressions in those languages, they attempt to determine whether or not it is possible to confirm the presence of a lexical base system, or compositional anchors, within these languages. Given that these expressions are not conventionalized and that their literal meaning does not seem to be numerical, the authors argue that it is not clear that these can be considered to form a lexical numeral system, or even whether they qualify as number words, supporting the possibility that this is another anumeric language found in the Amazon region.

Bowern [24], too, focuses on languages whose numeral systems are restricted in extent (e.g. with no way to represent numbers larger than 10), but her aim is to explore the variety of ways in which number can be represented in language—not only lexically but also grammatically. To this end, she examines whether and how languages with both restricted and unrestricted systems mark plurality. The objective is to show that numerals from Australian languages with restricted systems can exhibit great variability in their functions.

Building on some of the data on such restricted systems, Verkerk and colleagues [25] take an explicitly diachronic perspective by modelling the evolution of system structure on phylogenetic trees for language families from two areas where decimal systems are rare: New Guinea and northern lowland South America. Most languages in these areas either have numeral systems that lack structural components altogether or systems with relatively small compositional anchors of size 2 and/or 5. This contribution also aims to reconstruct the presence and size of compositional anchors in the various proto-languages of these two areas and their most likely transitions through time, thereby showcasing how taking anchors of all sorts into consideration can pave the way for a more fine-grained investigation of the factors that drive their emergence and evolutionary trajectories. As one of these drivers might have been the coexistence of body-based systems, the article also highlights the need to take systems in other modalities and the wider cultural context into account for such reconstructions.

A second article engaging in large-scale comparison of systems—in this case lexical systems with a decimal base—is the contribution by Koile and Blasi [26]. In search of commonalities and their potential causes, they ask an original question concerning base transparency and language: do irregularities in lexical numeral systems follow the same pattern(s) across languages and language families, and if so, what generates those patterns? To answer this question, the authors survey 118 languages with decimal numeral systems from around the globe, analysing whether they exhibit irregularities and which of these are base-related. Their investigation shows that, in spite of surface differences, many languages cluster around the same mathematical and compositional structure, and that deviations from perfect decimal structure reveal patterns shaped by both history and cognitive constraints.

The third article in this line of investigation shifts focus from lexical systems to a much less well-explored representational format. Dudojć and colleagues’ [27] contribution is breaking new ground by offering the first ever comprehensive survey of body-based representations of numbers, compiled and categorized in the *BodyBase* database. The authors explore how typologies and terminological frameworks apply to this particular format, and what sort of format-specific issues are encountered when relating body-based numerals to numerals presented in other formats. Harnessing the over 800 body-based systems recorded in their database allows them to identify general patterns across systems. In so doing, this contribution provides perspective on compositionality and base structure in a representational format that does not get the attention it deserves, and reminds us how compositional principles and tools, such as bases and anchors, can take on many shapes.

### (c) Cognitive implications and cultural imprint

As pointed out in the contribution by Bender [28], bases and the way they are implemented have implications for the structure of the system itself, for how users represent and process information and for cultural conventions. Research into what bases are and how they are implemented around the world will, therefore, not only generate new insights into the history and potential origins of our numerical abilities: it can also inform us on how our minds work and how culture and the mind shape each other.

Sometimes, tools are honest about their innards (or lack thereof). In such cases, simply looking at a tool is enough to figure out how it functions. By looking at a knife, for instance, we can see its component parts and how these make it work. In other cases, however, things are more complicated. For most of us, while peering under the hood of a car reveals a jumble of wires and parts, merely seeing how everything is organized is not enough to allow understanding of the vehicle’s functioning. This type of *transparency* has implications for the study of distributed cognition, owing to the tight relation between how transparent a system is and its *representational effects* [29,30]. Representing numerical information explicitly, and hence making it available for direct perception (i.e. in a transparent manner), reduces cognitive load and thereby facilitates processing compared with an implicit (non-transparent) representation of the same information.

Such lack of transparency is a recurring topic in this theme issue: how much does the physical manifestation of a composite numeral reflect its compositional structure? Given the diversity of ways in which numeration systems can be structured, both across sensory modalities and within each modality via different representational vehicles and formats, the degree to which their compositional structure is reflected in how its signs are organized can vary wildly. But does this variation have on impact on how we think about numbers?

Holt & Barner [31] introduce a novel experimental paradigm that could shed light on potential developmental origins of base-related cognitive effects. Their work exploits numerals that are not base 10, presented in both transparent and non-transparent formats, to study the development of numerical abilities in children. Specifically, they investigate whether compositional anchors with lower numerical values make it easier to grasp syntactic rules for numeral combinations, given that encounters with lower bases entail different cognitive requirements from the base 10 system that is typically used in other paradigms. Their results suggest that making the compositional structure of composite numerals more salient can indeed help children acquire compositional principles.

The format and structure in which a base is presented will also affect how we process the numerical information they are used to convey. Neth & Payne [32] take a deep dive into this representational effect, presenting results from two studies they conducted focusing on how strategies of mental addition are affected by the numerical base. Their results show that the way Indo-Arabic digits are structured around their base means that multi-digit additions that cross decade lines are processed differently depending on whether or not they feature complements (i.e. additions with round sums). Such internal echoes of the external cognitive tools that we use show just how integrated and distributed our numerical abilities really are, and how the value of the base can influence how we process numerical information. This marks a significant contribution to grounding the concept of base—and cognitive tools for compositionality more generally—in properties of cognitive systems shared by all.

Göbel *et al*. [33], finally, review a large volume of data showing that lexical systems whose compositional rules are more base-transparent tend to be easier to learn than those with less transparent rules—and/or with fewer regularities to begin with. While transparency here is a system-internal feature (e.g. when number words like ‘eleven’ and ‘twelve’ do not disclose 10 as a base), irregularity arises from the parallel use of two systems—such as a lexical and a notational system—where the order of numerical parts is inverted in one compared with the other (as in ‘fourteen’ versus ‘14’). The authors conclude that although it is not easy to disentangle the impact of linguistic factors from that of cultural practices surrounding the learning of number words, the presence of explicit base-marking terms and generative transparent rules appears to facilitate the learning of numeral systems.

Of course, whether it be cars, knives or numeration systems, tools do not exist in isolation: they are there to be used. In the case of numeration systems, most people learn to master several tools, even if some of these differ in terms of base size. The final three articles tackle this cultural embedding of, and the ensuing cultural imprint on, our numerical tools.

Much like Göbel *et al.’s* [33] contribution, Bender [28] too explores what happens when using multiple systems in parallel, but with a stronger focus on the challenges faced when these systems employ different bases. Taking counting systems as a specific type of measurement systems, the article investigates the relation between bases and counting units, their implications both for individual cognition and cultural conventions and some of the actual practical issues raised by numerical tools that have very real impact in our daily lives. In analysing the consequences—both positive and negative—of combining systems with diverging bases, it also outlines some implications for the evolutionary history of such systems.

A second contribution devoted to the origins and cultural shaping of numerical tools is the article by Chemla [34]. She argues that the two types of place-value notation systems that are thought to have emerged in parallel in ancient Babylon and China are sufficiently different that they cannot have a common source, as some have hypothesized. A key piece of evidence given here is that the role played by the base in each type of notation is completely different. This highlights the fact that paying attention to bases can reveal new and important historical facts about the origins of numerical practices, exhibiting some of the ambition to push the boundaries of research in the study of numerical cognition.

The theme issue closes with a contribution by Parkinson-Coombs & Núñez [35], who take us into novel conceptual territory by questioning whether and to what degree the concept of a numeration base might have influenced the development of representations for non-integers. They offer a detailed summary of the centuries-long process that extended the representational scope of Indo-Arabic numerals from integers to non-integers in the Arab world as well as in Europe, illustrating how calculation was a key factor in shaping the cultural evolution of bases in mathematical contexts. They also remind us of the importance of questioning culture-specific habits and conventions so as to avoid conceptual blind spots that may tilt research into narrow interpretations of the data.

## 3. Scaling up

A major goal of this theme issue is to clarify what numerical bases *are* in order to clear the way for a more in-depth investigation into how they work, how they shape our numerical abilities and what that reveals about cognition, language and culture. The authors of the articles compiled here have all taken significant individual steps towards this goal, enabling future work to answer questions both old and new. Four major advances come into reach now, namely regarding conceptualization and typology, data analysis and hypothesis testing, theoretical modelling of origins of numerical cognition and a broader understanding of cognitive implications of compositional anchors.

For instance, the theoretical distinctions and visualization proposed in some of the contributions to this theme issue place us on more solid ground for complementing, extending and even re-evaluating typologies of numeration systems. Most existing typologies, with their exclusive focus on bases and sub-bases, were primarily tailored to capture notational systems (e.g. [7,8,30,36]; and see [6]) and they therefore have been difficult to apply to less regular numeral types such as lexical or body-based systems [27] or to mixed and hybrid systems [10,28].

With conceptual consistency and a comprehensive representational tool in place, we are now also in position for a meaningful re-evaluation of the available data. Notably, instead of lumping a large range of systems together under labels such as ‘quinary’ whenever the symbol for 5 is special, we can now clearly distinguish which of these systems use 5 as a simple compositional anchor, a subbase, or a proper base. Doing this consistently allows for comparisons of all systems and their properties across representational formats. For instance, some linguistic systems whose structure tended to be described as ‘base-5’ can now be understood as equivalent to ‘base-10’ or ‘base-20’ systems in other fields, with the construction base in linguistics being a stepping stone towards the numerical base in the notational tradition within cognitive science.

This, in turn, will have implications for theoretical models. The literature on numerical cognition is rife with claims on which base sizes are most common, leading to claims regarding the origins and evolution of numerical tools and practices. Arguably the most popular of these claims is that decimal (and so-called ‘quinary’) systems prevail because they are grounded in our hands having five fingers. While recent work on body-based representations has cast some doubt on the universality of finger counting [37,38], we only now have at our disposal both the conceptual framework and sufficient data to scrutinize such accounts and their claims about correlations and causation. Contributions to this theme issue add to this conversation by pooling cross-cultural data concerning the distribution of compositional bases and anchors across the globe, in and across both lexical and body-based formats (e.g. [25,27]). Future work can follow-up on the data presented here to evaluate whether the prevalence of base 10 in today’s languages is owing to inheritance from a few joint ancestor languages that happened to use base 10, or whether it is a cultural attractor to which an initial range of diverse systems has converged over time.

On another front, while current cognitive models tend to be firmly rooted in empirical data, they are also compromised by the fact that these data were collected in a highly restricted range of cultural samples, with a set of tasks relevant to those samples, rather than to humankind at large. For instance, the prominent *triple code model* [39,40] presupposes written notation as a key component of people’s mental toolkit for representing and processing numerical information. In view of the long history of numerical cognition in the absence of notation, it is unclear how this and similar models would capture numerical cognition more generally or how well they can model purely mental arithmetic (as discussed in [41]). As argued by Göbel *et al.* [33], existing models are also all working from a simplified portrait of human users—as monolingual speakers and users of a single, typically decimal system—and they hence remain largely silent on the role of the base (or bases) *per se*. The conceptual clarification and the availability of both in-depth and large-scale data provided in this special issue, combined with theoretical discussion of bases and their interactions, means we are in an opportune position to take a new look at such theoretical models and update them to reflect more recent findings.

The role played by a base in the evolution and development of our arithmetical abilities cannot be overstated, as pointed out by Conant more than a hundred years ago. Without a base to anchor the composition of our representations of numbers, we would most likely never have managed to progress beyond the limits of working memory. Bases somehow integrate internal and external cognitive resources and allow us to count as high as we want (ideally).

In linguistics, taking a closer look at bases and related types of anchors can help to identify historical relationships between languages and establish contact points between cultures. In other domains of cognitive science, interferences between bases can show the inner working of the mind, as can the study of the rules underlying a perfect base system and how these compare with its irregular incarnation in a real human base system. In short, we have the potential to learn more about cognition, more about evolution, and more about the history of mathematics, culture and language, to name a few of those domains where studying the properties of bases promises interesting findings. Indeed, the study of bases has so much potential in so many domains that it is a wonder no one has thought about doing it—until now.

## Data Availability

This article has no additional data.
